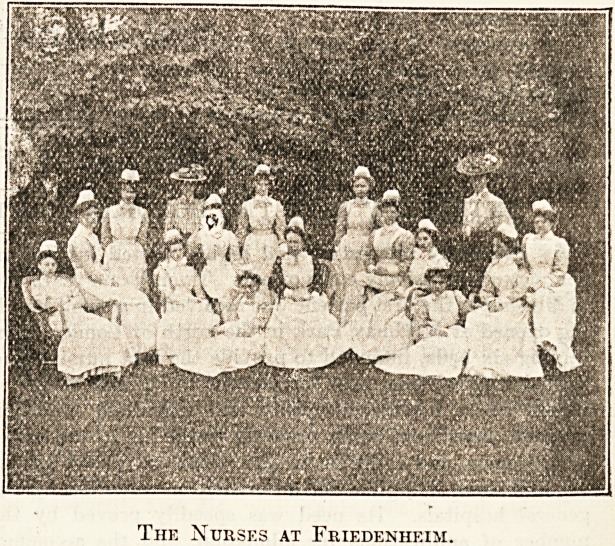# The Hospital. Nursing Section

**Published:** 1906-12-29

**Authors:** 


### The Hospital.

### Nursing Section.

Contributions for this Section of "THE HOSPITAL" should be addressed to the EDITOR, "THE HOSPITAL" NURSING SECTION, 28 & 29 Southampton Street, Strand, London, W.C.

No. 1,059.—VOL. XLI. SATURDAY, DECEMBER 29, 1906.

### Notes on News from the Nursing World.

### A COMPETITION FOR CHRISTMAS, 1907.

THE success of our experiment last year in inviting nurses to write our Christmas Supplement induces us to afford them the opportunity next year of contributing to our columns a special short story bearing upon hospital, infirmary, mental asylum, or district or private nursing life, not necessarily consisting of actual facts, but, if possible, founded upon them. The length of the story, which must, of course, be interesting and probable from first to last, should be about 5,000 words, and, other things being equal, preference will be given to the contribution which is accompanied by two or three good photographs or drawings, for purposes of illustration. The sum offered for the story is £5 5s., and the competition is open to nurses in all parts of the world. In order to stimulate effort, we shall send a cheque for £2 2s. to the author of the story which we consider second best, reserving the right to publish it. With the view of enabling our readers in the Far East and other distant quarters of the globe ample time to forward MS., contributions will be received up to June 30, 1907; but we shall be glad to have them as soon as convenient. They should be addressed to the Editor, Nursing Section of THE HOSPITAL, and marked outside "Christmas Competition."

### MR. SIMS AND THE DISTRICT NURSE.

IT is the practice of some people to paint the evil side of human nature in very gloomy colours, and no one can do this better than Mr. George Sims. He has the faculty of making the blood of his readers creep, and when he is writing fiction there is no particular reason why he should not give the rein to his vivid imagination. But when he deals with living people, even though they are supposed to belong to the criminal classes, he should lighten the picture and relieve its horrors. All is not dark, and in striking contrast to his lurid pictures of "life in shadow" in East London, with dangerous ruffians prowling round in search of prey, is the testimony of a district nurse, who, with 15 years of personal experience of night work, affirms that she has never yet been molested when walking about in Whitechapel, Spitalfields, Stepney, and Ratcliff. But in addition to this negative statement, she is able to speak of the respect, and even love, which is often shown by persons whom the sister describes as "creatures who are mere men" to nurses engaged in their duty among them in the long night watches, and to bear witness to the sympathy, the rough kindness, the self-sacrifice, and the care which they frequently manifest towards one another in sickness and trouble. Mr. Sims does not challenge her evidence, but replies that he was dealing with "the night duties of the police, and not with the experiences of nurses and priests." It can readily be understood that the attitude of the criminal classes to their natural enemies is very different from their behaviour to district nurses who may be of help to them, or to their own associates; and even these do not always come off scot free.

### A PRELIMINARY TRAINING SCHOOL.

AMONG the institutions where a young woman intending to be a nurse can commence her training at the age of 22 is Friedenheim Hospital. The superintendent of this institution, Miss Davidson, in conversation with our Commissioner, as reported on another page, gave some interesting particulars of the kind of experience obtained by probationers, and of the conditions under which they work. She does not gloss over the fact that the duties are arduous, and though alleviations are provided in the shape of considerable off-duty time, longer holidays than usual, and a very comfortable and well-appointed home, we can well understand that only one class can supply the right kind of nurse for such an institution. It is that class who take to nursing more as an avocation than as a profession, who combine general refinement of character with unaffected religious convictions. For these, whether as probationers or charge nurses, who, of course, are fully trained, there is ample scope at Friedenheim.

### INTEREST AND DISCIPLINE.

THE Wolverhampton Guardians were engaged at their last meeting in discussing a report respecting the nursing staff presented by the Nursing Committee. One portion of it dealt with the question of interest and the other with that of discipline. It was stated by the Committee that several probationers in the infirmary did not take sufficient interest in the lectures of the medical officer and the principal nurse, nor give sufficient time to study. On this point the Committee, having seen the probationers, were able to obtain assurances from them that no cause of complaint would arise in the future. The other part of the report had reference to allegations that one of the probationers had neglected to sign the book when she finished her duties 11 out of 14 times, that she returned on duty late, made tea while on duty, left her ward unattended, and absented herself from the lectures. The Committee therefore recommended that she should be dismissed. The chairman, in moving the adoption of the report, insisted upon the importance of maintaining discipline, and said that neglect of duty by nurses might mean loss of life. In the end the more merciful alternative of accepting the resignation of the probationer, which had been sent in owing to the report, was adopted. We have only to say that young women who do not feel that they can take an interest in their work are quite as unfitted to engage in nursing as those who cannot submit to necessary discipline.

### SHEFFIELD QUEEN'S NURSES AND THE HOSPITAL SUNDAY FUND.

Considerable dissatisfaction has been created in Sheffield by the refusal of the Hospital Sunday Committee to admit the Sheffield branch of the Jubilee Institute to a share in the Fund. The decision is the more surprising because the Council of the London Hospital Sunday Fund came to a contrary conclusion in the spring of this year, and the Jubilee Institute in London accordingly received a portion of the money collected. We think that there are two good reasons why the Sheffield Committee would do well to reconsider their determination. One is, of course, that we regard the claims of the local branch of the Jubilee Institute on the Fund as incontestable; and the other that the Fund itself is likely to suffer from persistence in a policy of exclusion.

### PROBATIONERS AND EXPENSIVE MEDALS.

After considering the question on two occasions the Guardians of the City of London have finally decided to have three medals struck at a cost of £6 to present to the first three probationers passing their examination. We appreciate the motive of the Guardians, but we think that a more practical gift obtainable for a smaller outlay would be more useful to the probationers themselves, and would be less open to criticism outside.

### A COMPROMISE AT ROTHERHAM.

The Rotherham Guardians having signified that they do not desire the infirmary to be under separate administration from the Workhouse, but wish the superintendent nurse to have more responsibility in regard to the management, the Local Government Board have intimated their readiness to issue an order similar to that issued last July to the Chelmsford Guardians, and now in operation. Under this order the entire administration of the infirmary buildings is placed in the hands of the superintendent nurse, "subject to the general superintendence of the master." The Rotherham Guardians have agreed to ask for a similar order.

### THE PERIL OF UNCLEAN CLOTHING.

The question of unclean clothing has been raised in a special form in Lancashire. At an inquest at Accrington on the body of a miner who died from blood-poisoning, Dr. Clegg pointed out the peril of unclean clothing to which colliers are especially exposed. When they suffer an injury the blood and matter get into their underclothing, and if they happen to receive another wound there is a danger of it becoming infected by the poisonous germs upon the clothes. Dr. Clegg urged, in their own interest, that they should wear garments that can be washed every week, instead of using old clothes that are never washed. The importance of this advice is obvious, and we think that nurses who work in mining districts might perhaps do more to bring it home, not only to the men themselves, but also to their womenkind, by taking advantage of any opportunities that offer to discuss the matter and try and suggest a remedy.

### RESIGNATIONS AND DIETARY.

It is distinctly detrimental to an institution when reports are circulated that the dietary is bad. Three nurses have recently resigned their posts at York Workhouse, and a rumour obtained currency that their action was due to bad dietary. At the last meeting of the York Guardians it was stated that the resignations were owing to other causes, but as one of the Guardians subsequently said that "the diet had been revised in order to get a greater variety for the officials, and was now as good as it was in any union," it can easily be understood how an erroneous impression obtained credence. We are glad to learn that now, at all events, the dietary is satisfactory.

### THE DEATH OF A NURSE IN A CONVENT.

The interest of a story told in the early part of last week before the Deputy-Coroner at Kensington at the inquiry into the death of Miss Emily Tibworth, was accentuated by the details given on Friday by a friend of the deceased. Miss Tibworth was a certified nurse who was called into the Roman Catholic Convent of Poor Clares in Cornwall Road, Notting Hill, to assist in nursing some of the nuns, who were ill with influenza. She became so attached to the cause that she proposed to enter the seclusion of the order of the convent. She passed through certain preparatory stages, but the mother abbess then came to the conclusion that she was not fitted for the order, a decision which caused her great distress, and prompted her to threaten to end her life. The day after she was found in her cell dead in her bed with her arms across her breast and a crucifix in her hand. The medical evidence proved that death was due to syncope from rupture of the heart, and there was no sign of any poison. Moreover, as her friend, Miss Pauline Willis, subsequently pointed out, the fact that the funeral was preceded by a solemn service in the convent chapel, and the remains were followed to the grave by some of the nuns, further disposes of the idea of suicide. The case is a very sad one, but we can no more blame the mother abbess for refusing to allow Miss Tibworth to join the order after serving as a novice, than we should blame the matron of a training school for declining to admit as probationer a candidate who in her trial months had shown that she was not suitable for the work.

### CENTRAL MIDWIVES BOARD EXAMINATION.

The number of candidates examined by the Central Midwives Board on December 12 was 208, and of these 153 passed, the percentage of failures being 26.4. No fewer than 27 of the successful candidates were trained at the Maternity Charity,

Plaistow, the next largest number being 11 at the Salvation Maternity Hospital. The remainder included nine trained at Queen Charlotte's Hospital; eight each at the City of London Lying-in Hospital and Clapham Maternity Hospital; six at the East End Mothers' Home; five at the British Lying-in Hospital; four at the Brighton and Hove Hospital for Women; three each at the Rotunda Hospital, Dublin, and the General Lying-in Hospital; two each at Guy's Institution, the London Hospital, British Royal Infirmary, Dundee Maternity Hospital, West Ham Union Infirmary, and Liverpool Lying-in Hospital; and one each at Kensington Union Infirmary and Greenwich Union Infirmary.

### THE SOCIAL UNION IN SOMERSETSHIRE.

Meetings of the Nurses' Social Union have been held recently at Wells, Yeovil, and Taunton. At Wells the meeting took place at the Bishop's Palace, and the nurses present will not easily forget the welcome given them by Mrs. Kennion and the kindly sympathy and counsel of her little address. A lecture was delivered by Miss Fergusson, Organising Inspector of Domestic Subjects under the County Council, on the way in which nurses might help to strengthen the hands of those who were trying to spread a knowledge of hygiene and wholesome living amongst the people. The guests were shown over the palace, and tea in the crypt introduced quite an element of romance to that meal. At Yeovil Dr. Kingston gave an absorbingly interesting lecture on th *x*-rays, illustrated by magic lantern slides. Mrs. Maxwell kindly entertained the Taunton Branch at her house, and a large audience were assembled to enjoy Dr. Hogg's lecture on microbes. It was thoroughly appreciated, and tea and music ended a successful meeting. The loan collection as usual proved a centre of attraction, especially as some additions have been made, which were novelties to most of the visitors.

### EXTENDED FACILITIES AT EXETER HOSPITAL.

Last week we published the names of the successful probationers at the annual examination conducted at the Royal Devon and Exeter Hospital. On the afternoon of Thursday, the 20th, Lady Iddesleigh attended in order to present the medals and prizes. The President, in proposing a vote of thanks to her, referred to the great help which the nurses will have in the studies of the coming year in consequence of extended facilities being available in the operating theatre. Before long, he added, there is also to be an electrical apparatus which, he claims, will make the hospital unique in the West of England.

### A DEAN'S VIEW OF THE NURSING PROFESSION.

At the annual meeting of the Manchester and Salford Sick Poor and Private Nursing Institution, the announcement was made that the receipts for the year fell short of the expenses to the extent of £300. The Mayor of Salford, who was in the chair, the Bishop of Salford, and the Dean of Manchester pleaded vigorously on behalf of the organisation, whose nurses attended 10,000 people and paid a quarter of a million visits during the twelve months. The Dean having described the Association as one of the subterranean charities of the city, which does not attract notice by external signs, possesses no great building, and is not advertised by means of any flaming posters, continued: "As to the nurses, he had to say that if there is a sacred profession in the world it is the profession of nursing. It is in times of sickness and suffering that womanhood find their best opportunity of serving humanity." Miss Amy Hughes had the pleasure of being on the platform with Dean Welldon and hearing him pay this striking testimony to the workers whom she represented on the occasion.

### THE LONDON BIBLEWOMEN AND NURSES' MISSION.

There has just been published, by Messrs. J. M. Dent and Co., a little book from the pen of Miss Rose E. Selfe, which, while confessed a record of the work of the London Biblewomen and Nurses' Mission during its 50 years of existence, throws light on many matters of interest to nurses. The development of district nursing, and the training requisite for such work, are among other topics dealt with. But above all, the author of "Light amid London Shadows" has striven to set before her readers the bond of human love and mercy and its claims on rich and poor alike, which this season so unfailingly demonstrates. Many perusing this little book, whether they be nurses or not, will take heart of grace from its pages telling of difficulties met and overcome, and begin the New Year with fresh courage and perhaps new ideas.

### QUEEN'S NURSES AT LOUGHBOROUGH.

An extremely satisfactory report was presented at the annual meeting of the Loughborough Queen's Nursing Association, showing that between five and six thousand visits were paid by the nurses in addition to 100 casual visits. The number of patients nursed was 171. Both in patients and in visits there was an increase upon the previous year, and it looks as if the Association would shortly require to appoint another nurse. Happily, the receipts for the last twelve months exceeded the payments by £38, and as the work of the organisation appears to be done in a most vigorous and businesslike manner by entirely unpaid officials, we do not anticipate that much difficulty will be experienced in obtaining sufficient additional support to justify an extension of operations.

### DOOR-TO-DOOR COLLECTIONS.

A feature in connection with the working of the Gateshead Nursing Association last year was the organisation of a door-to-door collection in certain districts. This was described at the annual meeting as a very arduous way of obtaining money, but the Chairman said that it was really the only way to secure a large number of small subscribers. The success of the innovation at Gateshead is proved by the fact that £35 12s. 8d. was obtained in this manner, thanks to the ladies who carried through the task. We commend this result to other nursing associations in greater need than that at Gateshead, who are now in the satisfactory position of possessing a balance of £168 in hand.

### The Nursing Outlook.

"From magnanimity, all fears above;
From nobler recompense, above applause,
Which owes to man's short outlook all its charm."

### NURSE TRAINING AND HOSPITAL EXPENDITURE

**"The attention of the committee has been called to the extensive arrangements for teaching nursing at hospitals, and while not wishing in any way to disparage work so obviously advantageous to the public, it may become necessary to consider to what extent expenditure is incurred for nursing beyond the requirements of the nursing service of each hospital."**

We give prominence to the above extract from the report of the Hospitals Distribution Committee of King Edward's Hospital Fund for London for 1906 because of its importance. Those managers of hospitals who keep in close touch with the cost at which the hospitals are conducted and the progress made each year in this direction, will notice, if they refer to the volumes of Burdett's "Hospitals and Charities" for the last few years, that the cost of nursing is steadily increasing. An examination of the figures will bring out the further fact, that, the number of nurses, or rather of probationers, employed, each year, in many hospitals with nurse training schools, is increasing too. It has already been decided, after full inquiry by a special committee, that no portion of the hospital funds given for the maintenance and treatment of the sick poor, may be employed for the purposes of medical education. In such circumstances it becomes a question for consideration whether it is legally justifiable to devote any such funds to the training of nurses beyond those who are required for the purposes of each hospital's necessary work.

There is another and even graver question which affects mainly lying-in hospitals. That is, the training of midwives and monthly nurses, who are not required for attendance upon the patients, in the particular establishments where they undergo their training. Midwifery pupils, as a rule, pay fees for their instruction, and it is held, that, those fees should include a sum, which will defray the whole cost to the hospital of their training, and any other expenses incurred by the hospital in regard to such women.

King Edward's Hospital Fund, in addition to the splendid work it has accomplished in making the voluntary support of hospitals, adequate, so far as London is concerned, has accomplished an equally great work, as pointed out by the Prince of Wales, by the issue of a statistical report on hospital expenditure and prices, which now includes forty-eight hospitals. These reports, commencing in 1904 with only sixteen hospitals, have had the effect of showing, as H.R.H. stated, "a large reduction of expenditure last year, the value of which, taking into consideration the increase in the number of beds, is equal to a saving of about £20,000." The Prince added: "When the other hospitals have had time to take advantage of the information which our reports provide, we hope this satisfactory saving will be largely increased." Another result of this action of the King's Fund has been most gratifying, from the fact, that, many of the hospitals have readily seconded the Prince's efforts by the appointment of economy committees. This question of the cost of nursing and all that relates to it is one which we hope each economy committee will carefully investigate and consider. We anticipate, if this course is adopted by the majority of hospitals with nurse-training schools, that they will assimilate their system to one which some institutions adopt in America, where probationers are taken for training, in addition to the actual number required to do the work of the hospital, up to the limits of the accommodation in the nurses' homes, on the payment of a reasonable fee. Economical administration in the case of a voluntary hospital means increased contributions from the intelligent givers to hospitals, who are more and more coming to understand the importance of economy, and to support it, where they find it, by increased contributions. It follows, that, the appointment of these committees of economy may prove of great pecuniary value to each institution, not only by regulating and reducing expenditure to an average and reasonable sum each year, but by attracting new and important subscribers, who must strengthen the resources of each hospital which secures their steady support.

Of late years there has been a marked change in the methods of the training schools in regard to the selection of probationers. In all well-managed schools the matron's aim has been to secure candidates of education, intelligence and character. They are drawn largely from classes of the community, who could and ought to pay reasonable fees, for instruction in nursing. This fact is material in considering the question of how far and to what extent the hospitals should afford facilities for training nurses, other than those which may be required to carry on the work of the particular hospital. It is obviously open to question to devote money, given by the public for the care and maintenance of the sick, to the training of such extra probationers. When, however, a reasonable fee is forthcoming for their education, and there is no charge on the hospital revenues, the admission of extra probationers may be attended by many advantages. It may then become a legitimate portion of the work which a nurse-training school can properly undertake.

### Nursing in Tropical Climates.

By Andrew Duncan, M.D., B.S., M.R.C.P., F.R.C.S., Fellow King's College, Lecturer on Tropical Medicine at the London School of Tropical Medicine, and the Westminster Hospital.

### III.—PRICKLY HEAT.

Another affection for which heat is responsible is prickly heat. It is generally held to be a minor ill, and moreover the unfortunate sufferer from it is considered to be lucky in that he is less liable to the more severe diseases of the tropics. This, however, is poor consolation at the time to one experiencing the maddening irritation induced by the condition, for the distress may be so acute that the term "maddening" can in truth be only applied to it. It is also one usually considered a minor ailment, not causing anxiety, so that very often a doctor is not called in. Hence it is well to know the best means of allaying the intense irritation, and a nurse may thus render valuable comfort to the sufferer.

*Nature of the Affection.*

The skin amongst other structures contains two sets of glands, the sweat glands and the sebaceous. Many authorities hold that it is directly connected with the former, but Pearse of Bombay dissents from this view, and considers the latter are the culprits. His view is that there is an acute distension of the sebaceous glands by their own secretion primarily, which is maintained in a more or less acute and active state by the continued irritation of excessive perspiration, and his treatment, which certainly seems to be more successful than the usual methods, is based on this view. It has, as usual with a number of tropical diseases, been asserted to be a form of malaria; but, of course, it is not.

*Features of the Affection.*

New arrivals are most liable to the affection: it will very likely be first experienced in the Red Sea on the voyage out to India. The eruption consists of small bright red pointed discrete papules, placed close together, mingled with a few vesicles. It occurs most on the front of the chest and abdomen, the back, shoulders, and arms, and less often on the face and legs. Pearse points out that it is distinctly limited to those parts of the skin containing sebaceous fluids. Large areas of the body are affected. The rash appears quickly, with profuse sweating. The itching is intolerable, and provokes much bad language! Often one sees a very fine desquamation superseding on the parts. Occasionally the itching is so intense that sleep may be disturbed. The duration is from a few days to months; should a few days of cooler weather intervene, relief will occur.

*Treatment.*

(*a*) *Prophylactic.*—As has been remarked above the affection is considered a minor one, the doctor may not be called in. It is well therefore that the nurse should know what to advise if asked to do so. First of all, anything exciting perspiration, whether we consider this as causing the affection, or as keeping it up, must be avoided. Thus all hot drinks should be eschewed, such as a cup of tea, which, in my experience, is one of the surest things to bring on an attack on newcomers. Close rooms are contra-indicated. As regards the clothing, Pearse advises cotton next the skin, and recommends an openwork cotton. Here, however, I feel bound to dissent from him. It has before been shown that cotton should not be worn next the skin. This becomes sodden with perspiration, and is in my experience in no way any precaution against prickly heat. Light woollen merino is the best to wear, this absorbs the perspiration quickly, so that the skin never becomes sodden.

(*b*) *Curative.*—Supposing a medical man be not called in, it may very often happen that a nurse may be in the bungalow, etc., and be asked her advice as to alleviating the intense itching. Various remedies have been advised, but in my experience none can equal bathing the irritated parts of the body with water containing "washing soda." I had been recommended to try many applications, but the latter was the only one that effected any relief to me. This was, however, before the appearance of Dr. Pearse's paper on the subject, and the method he recommends appears to be the best hitherto advocated. It will not, as he says, cure; for if you leave off the treatment the disease will return in a day or two, but it is a means of affording the very greatest relief. The nurse should therefore advise the patient, in cases where her advice is sought, to anoint the body with oil for the purpose of protecting it against the irritation of the exuded sweat. This also keeps the skin soft. Various preparations can be used. Pearse recommends a mixture of almond oil and lanoline in the proportion of 8 to 1, with ol. rosæ. Major Moore, R.A.M.C., has used with great efficacy fresh cocoanut oil. Most people in the tropics look upon this as a nasty product, but if fresh it has only a very faint smell, which disappears when it is rubbed into the skin. It is recommended to apply it before going out for the evening's exercise. You strip, pour a little oil into the palm of the hand, and rub it over the body from the neck to the ankles, getting your servant to do the back. Do not use a sponge or rag, as they are not easily cleaned and soon get offensive (Moore). Soap should be avoided, as "this removes the sebaceous matter from the surface of the skin, the sebaceous glands are thus unduly stimulated to produce more secretion, while at the same time the excessive perspiration is also irritating them to lubricate the surface" (Pearse). As a further aid, dusting powder is most valuable. When no other is handy fine Indian corn meal can be applied.

In concluding the account of these methods of warding off prickly heat, I must state that I have had no personal experience of the latter procedure, as it had not been advocated when I left India, but there can be little doubt that these oily preparations constitute the best means of preventing the distressing attacks of this affection.

### Communicable Diseases.

We now must consider the nursing of those diseases in the tropics that are communicable from one patient to another. This process of communication is due to the agency of the lower forms of animal and vegetable life—for example, amongst the latter we have the germs of typhoid fever, of relapsing fever, of cholera; amongst the former we have the amœba of dysentery, the trypanosoma of sleeping sickness, and the sporozoa of malarial fever. We may profitably begin this part of our subject therefore with a short account of the life history of germs.

*Germs.*—These may be either small spheres, when they are known as "cocci," or may be straight or curved, when they are known as "bacilli." A third class is that of the wavy filaments, classified as "spirilla." Under the microscope they may be seen scattered without any definite formation, or arranged in pairs (diplococci), or in long chains (streptococci), or in collected masses (staphylococci).

*Mode of Growth.*—These minute organisms propagate themselves in the following ways: in one method a division occurs along the middle, so that the original germ separates into two, and these again subdivide, each subdivision becoming a separate organism, as is shown in the following diagram:—

The process is a very rapid one. Cohn states that in twenty-four hours a single organism may produce a progeny of sixteen millions. Another method is by spore-formation. Here small rounded spots appear in the germ, which then breaks up and liberates the spores, each one of which becomes a new organism.

*How do the Organisms Produce Disease?*—The healthy tissues of the body are able to offer great resistance to the attacks of these minute forms of life, but only a slight chemical or physical change is necessary in the vitality of a part to lay it open to attack, and a germ can then cause disease in the following ways:

1. Mechanically by obstructing the performance of the different functions of the tissue.
2. In their growth by depriving the tissues of oxygen.
3. By acting as a poison to the tissues.
4. By producing poisons or toxines.

The body itself possesses the power of counteracting the attacks of these germs, in that the blood contains a certain set of cells termed "leucocytes," which have amongst their properties the valuable one of overcoming these enemies to the health of the individuals. Should, however, the germs be in excessive number, and potent enough to overcome their resistance, then the body will become infected. The resistance of the body to the action of germs varies with the individuals; some people pass unscathed through an epidemic, others soon fall victims. The resistance to the various diseases is known as "immunity."

Now many conditions will either increase or diminish the potency of these germs to do evil. Of late years methods have been discovered by which the vicious qualities of the organisms can be so modified as to render them capable of neutralising the attacks of the same when the human body is exposed to them; in fact, antitoxins are formed. The antitoxin treatment, as we shall see, is of the greatest value as regards prophylaxis in cholera and enteric fever.

*How do Germs Enter the Body and Infect the Individual?*—Unfortunately manifold are the paths of entry of these organisms into the human frame. To mention a few of them, they can infect by the food and liquids we swallow or by the air we breathe. They can be conveyed thus by infection from the excreta, fæcal and urinary; by the tropical dust; they can be conveyed by infected clothes, bedding; whilst a notable instance, as will be shown later, demonstrates that they can gain admission through the medium of jairans" or dusters. It is thus seen how necessary in the preventive treatment of heat disease are well considered plans of disinfection.

### The Nurses' Clinic.

### INTERNAL INJURIES AND POISONOUS BITES.

*Contusion of the Lung.*—This is not an uncommon complication of fractured ribs. In severe cases there is difficulty in breathing, and after some days the patient will cough up a large quantity of dark viscid sputum, and as this is got rid of the difficulty in breathing ceases and the patient recovers. Absolute quiet in bed must be maintained, the patient being propped up with pillows or a bed-rest. The room should be kept warm and well ventilated, light diet given, and the bowels regularly opened. Internally the doctor will probably order expectorants, to hasten the clearing up of the lung. In perforating wounds of the chest it should always be remembered that patients should be kept lying on the injured side, to secure more rest for that side, unless, of course, the surgeon should give orders to the contrary.

*Wounds of the heart* are mostly fatal, but not always so; in several cases the wound has been sutured, and the patient recovered.

In nursing these cases absolute quiet and rest must be maintained, and the patient guarded against any sudden shock or noise. As the patient will be in a very feeble and nervous condition owing to the shock and loss of blood, there is great danger of pericarditis setting in. No one but the doctor and nurses on duty should be allowed in the sick-room.

*Rupture of the Stomach and Intestines.*—These cases will probably be operated on immediately, even should there be a considerable amount of shock, as prompt measures may be the only means of saving the patient's life. Laparotomy will be performed, and for ruptured intestines enteroraphy may be resorted to. These cases are treated as ordinary abdominal operations, but if a Murphy's button has been used to join the two ends of the intestine, the nurse must

### THE NURSES' CLINIC.—*Continued.*

watch and see if it passes out with a stool. Sometimes it may remain in the bowel, causing obstruction and perforation, and the surgeon will rely on the nurse to know whether it has passed out.

*Rupture of the Liver.*—The patient must be kept absolutely quiet, and no brandy or stimulants given unless ordered, for fear of increasing the hæmorrhage. Should stimulation be ordered, the nurse should have the hypodermic syringe with strychnine ready, as it will probably be ordered. If there is evidence of bleeding, the patient will have to be prepared for laparotomy, which will be nursed in the usual way.

*Fracture of the Pelvis.*—The symptoms are great pain low down in the abdomen, greatly increased by movement, and the patient cannot stand, but complains of "falling all to pieces." He should be laid on his back in bed—a fracture bed if possible. The nurse must try and prevent the patient from passing urine till the surgeon has seen him, as there may be injury to the urethra, from which, if injured, blood will trickle. If the bladder is injured there will be inability to pass urine, and when the catheter is passed only a small quantity of blood-stained fluid will come away. A broad flannel bandage will be bound round the pelvis, or a belt. As the patient will be kept in bed for about eight weeks, the same care will be required as for a spinal injury. It will be more difficult to keep the back clean and dry, owing to the flannel bandage. For the first four or five days the bowels are kept freely open and the bladder emptied frequently, to prevent abdominal distension. After eight weeks the patient will be allowed to get about with crutches.

*Rupture of the Kidney.*—The symptoms are severe shock, with pain shooting down the thigh and blood in the urine. The patient must be kept strictly quiet in bed, and if in pain will probably be ordered morphia, as the suffering is very great at times. Food should be given by mouth, in very small quantities frequently. Bandaging the abdomen will sometimes give relief to the pain. The bladder will be washed out periodically, and the nurse should always have a couple of large-eyed catheters ready sterilised for use. If hæmorrhage persists the surgeon will probably cut down and remove the kidney, if necessary.

*Stings of Insects in the Mouth.*—These are frequently caused by wasps and bees. They cause acute pain and rapid swelling of the tongue, which may be bad enough to threaten asphyxia and need cutting. More generally, however, the swelling subsides in a few hours and disappears. The best treatment is to wash the mouth out constantly with a solution of bicarbonate of potash or soda, which neutralises the poison.

*Snake Bites.*—Prompt action only is of use in cases of snake bite. Immediate application of a tight bandage above the wound, tight enough to arrest all circulation, and thereby prevent the poison entering the system. Freely excise the part, or if it is a finger amputate at once; if no doctor is present even, an attempt should be made to cut the finger off. Men have been known to bite or chop their own finger off; this is far the most effective treatment when it is possible. Cauterising may be done by any means at hand—a red-hot poker, iron, nail, nitrate of silver, or by making a paste of gunpowder and setting it alight, allowing it to burn out the part. Sucking the part is of use, provided it can be kept up long enough. Permanganate of potash, if obtainable, after excision should be freely applied to the raw surface. This is supposed to destroy the poison by oxidation.

Collapse must be treated by stimulants—brandy, whisky, or ammonia, very strong. The patient must be kept walking up and down as long as possible, and then artificial respiration resorted to when he has difficulty in breathing. Should the patient live, his strength should be kept up by milk, eggs, beef-tea, soups, and stimulants, frequently administered in small quantities. Some doctors have injected large doses (10 to 20 minims) of liquor strychnine with favourable results.

As most of these cases will prove fatal, it is advisable to try any and every remedy at hand, as something may help and nothing can make matters worse, but remember that "desperate cases need desperate remedies."

### Incidents in a Nurse's Life.

### THE CHRISTMAS BURGLAR.

Christmas Eve was fast drawing to a close. In a few minutes the clocks would chime the midnight hour, and another Christmas Day would be born.

In our provincial hospital all was quiet and peaceful.

The night nurses were mostly engaged in preparing their midnight meals. There were no critical cases in the wards, on which fact each one was inwardly congratulating herself. The lights burnt low in wards and corridors; the fires gave out a cheerful glow, showing up the bunches of holly and trails of ivy which helped to form the Christmas decoration. The night nurse of the male surgical ward was giving a final polish to the taps in the surgery, which was one of her nightly duties, before sitting down to her meal. The house-surgeon had done his night round, but had not yet retired to bed. In her sitting-room, night-sister was waiting for the clocks to strike, then she would go round and wish each nurse a Happy Christmas.

All at once the peace was broken by a crash which resounded through the whole building. It sounded as though some crockery had been thrown violently to the ground, and in the quiet of the night the noise was doubly intensified.

The house-surgeon stepped briskly out of his room and into the surgery, a look of annoyance on his face, where nurse, her polishing suddenly suspended, was gazing with wide-eyed amazement into space.

"What do you mean, nurse, by making such an unearthly row at this time of night?" he snapped out.

Nurse was immediately alert and on the defensive.

"I have not been making a noise; that crash was in the basement, or so it seemed to me."

"I'm sorry, nurse!" He was humble at once. "Please go to night-sister, and ask her to find out the cause of the noise." He strode back to his room and shut the door.

Night-sister was already half-way to the surgery when nurse met her, and immediately began, in a querulous tone: "Nurse, I do wish you would not drop things, you will have wakened everyone in the place."

"It was not I, sister; the crash seemed to come from the basement, and Mr. —— wants you to find out what it is."

Nurse's voice had by this time assumed a grieved tone. She knew she had earned a certain notoriety for noise, but to be twice accused of making a disturbance she was innocent of was distinctly hard, and just as she was feeling so happy about Christmas, too! The clocks had in the meantime chimed the midnight hour.

"I'm so sorry, nurse!" Sister's voice also sounded grieved. She had been on the point of starting on her round of greeting.

"Where is Mr.——?" she questioned.

"Gone back to his room," was the quiet reply.

"Gone back to his room—well, I do think he might——"

Sister stopped short, and did not finish her sentence. The corners of nurse's mouth twitched, though she resolutely suppressed the smile which threatened to overspread her features. She was beginning to enjoy the break in the monotony, though she still felt badly treated.

"Get your lamp, nurse," sister continued; "we will go to the basement and investigate."

The Advance of the House Surgeon.

Nurse brought the lamp, and together they started, but just at that moment the house-surgeon again came out of his room, this time armed with a stick.

"I will come down with you, sister," he said, as he joined them; "there are so many unemployed about the town, and with so much extra food and other things about it is——"

His voice died away, as he took the lamp from nurse, and passed ahead of them.

"I wondered if you intended us——" sister began, but stopped short again, and nurse, bringing up the rear, smiled broadly to herself.

"We will go to the kitchen first," said the house-surgeon, not noticing the interruption.

Nothing was found out of place there. Along the dark corridor they went, the house-surgeon heading the procession with the lamp and stick, peering into every corner, and prodding every likely hiding-place. The boot-room, chip-house, coal-cellar, were visited in turn, but nothing wrong could they find. The scullery came next, and just as they were about to enter it, there came a noise from the coal-cellar as of sliding coal.

"Must be something there!" muttered the house-surgeon, again heading for the coal-cellar.

The two ladies began to feel an uncomfortable sensation creep along their spines, and the amused look faded from nurse's face. They kept at the heels of their leader, and they both scarcely dared to think what was going to happen next. Nurse gave a little gasp as the light was flashed round the coal-cellar, and then heaved a heavy sigh of relief. There was nothing to be seen except coal.

"Strange," muttered the house-surgeon.

"The noise certainly came from here," said sister.

They retraced their steps to the scullery, and the house-surgeon flashed round the lamp at arm's length. There on the stone floor lay a large enamelled dish, and the broken pieces of a plate, and close by a piece of cold fish.

The crash was explained!

The burglar was a cat, which must have found its way in through the coal-cellar, and, the door of the scullery being open, she had jumped upon the shelf, bringing down dish and all.

This was the decision arrived at, though they could not see the animal, which they decided must have slipped past them in the gloom, and escaped by way of the coal-cellar, thus accounting for the sliding of the coal.

"I am glad you had not gone to bed," said sister to the house-surgeon, when they had regained the upper regions. "We should have been nervous about coming down here alone after that awful noise." She had been inclined to misjudge him at the outset, and was anxious to make amends.

"Poor nurse here got the blame for making it," she continued, smiling.

"Yes, nurse scored that time," said the house-surgeon, with a humorous twinkle in his eye, as he shook hands with them, and wished them—as I now wish you all—"A Happy Christmas."

### The Nurses of Friedenheim Hospital.

INTERVIEW WITH THE HONORARY SUPERINTENDENT. BY OUR COMMISSIONER.

An extremely interesting and highly valued institution has just unobtrusively come of age. Twenty-one years ago in November the first patient was received in a small building opened at Mildmay Park in the north of London, containing six beds, intended to provide the best nursing and skilled medical treatment for persons in the last stages of illness where insufficient means and friendless condition prevent them from being properly cared for to the end—men, women, and children, in fact, whose advanced disease renders them ineligible for admission or retention by the general hospitals. Its need was speedily proved by the number of applications for admission, and the accommodation was soon increased to ten beds. At the end of seven years it became obvious that a much larger building was urgently wanted, and in November 1892 the present Friedenheim Hospital in Upper Avenue Road, Swiss Cottage, was opened by the late Duchess of Teck. Thus the experiment of Miss Davidson, the originator and founder of the now widely known Home of Peace, who subsequently was cordially assisted by Dr. A. T. Schofield and other friends developed into a notable permanent charity. But Miss Davidson, when I visited Friedenheim the other day, in order to ask her about the work of the nursing staff, wished me to keep her efforts in the background, and it need only be added that from the outset until now she has acted as honorary lady superintendent, or, to put it in her own words, "mother of the home."

"For the nursing and housekeeping arrangements, however," said Miss Davidson, as she showed me over the wards, "the matron, Miss Meldrum, is responsible. But, of course, I know all that is going on, and, except during a short period of the year, I am always here."

"How long has Miss Meldrum been matron?"

"Five years. She was trained at the London Hospital, and was afterwards night superintendent at the Bristol Royal Infirmary. As you see, the wards are on three floors, and there is a charge nurse for each floor, and one who acts as night superintendent with three probationers under her.

### THE NURSES OF FRIEDENHEIM HOSPITAL—*Continued.*

The number of beds is 48, and during the day seven probationers are on duty. I try to have two probationers extra available for holiday duty and in case of emergency. Twelve are really required."

### The Training of Probationers.

"Are the charge nurses fully trained?"

"Occasionally a very capable probationer is promoted at the end of her term to be a staff nurse, but our rule is to

The Nurses' Home at Friedenheim.

have a fully-trained nurse from outside to act as charge nurse."

"What is the age of admission?"

"From 22 to 28. I think that it is rather hard for a girl who wants to be a nurse to have to wait until she is 24 to begin. Of course, our probationers usually go on to other hospitals for general training, but we start them here on their nursing career. They come for two years. The training consists of work in the wards, and lectures are given by Dr. Lush, the medical officer, and by the matron. At the end of the term a certificate to the effect that the probationer has performed her duties satisfactorily, and passed the prescribed examination in medical and surgical nursing, is signed by the consulting staff, the medical officer, and myself."

### Practical Experience.

"Do you care to say anything about the particular nature of their experience?"

"If they are young women who will live for the work they learn a great deal. They are taught to do much, and have many opportunities of applying splints and doing dressings. The cases are very varied and interesting. Cancer and consumption form the majority, but we take in patients suffering from all other diseases, unless they are infectious. We have operations performed for the relief of patients. Colotomy is frequently done here, and also tracheotomy. We are quite willing to receive, and able to nurse, surgical cases after operation when the patients are not likely to recover. Mr. Langton has just given us a very up-to-date operating-table and dressing-table. You saw some of the children enjoying a birthday tea in the ward, but the cases, chiefly heart or hip disease, and consumption, are supposed, like the others, to be in the advanced stages. A few beds in the 'Helena' wards are reserved for paying patients."

### The Time-table.

"What salary do the probationers receive?"

"Ten pounds each year. If they remain on as staff nurse, £20 the first year, £25 the second, and £28 the third. Probationers are required to wear uniform, which is given them, inside and outside; but the charge nurses can please themselves as to the latter. The day charge nurses are paid from £28 to £30, and the night charge nurse £33 to £35.

"Your time-table would be interesting."

"The day staff enter the wards at 7.30 A.M., after breakfast; lunch is at 9.30 to 10; dinner, 1 to 2.30 P.M.; tea, 4.30 to 5; prayers, 8.30 P.M.; supper, 8.45 P.M. The off-duty time is two and a half hours daily; half day, from 2 to 10, every fortnight. The night nurses have supper at 7.45 P.M.; are in the wards from 8.15 P.M. to 8.15 A.M.; prayers at 8.30 A.M.; breakfast, 8.45 A.M. They are off duty in winter from 9.15 to 12 A.M., and in summer from 5 to 7.45 P.M., going to bed in winter at 12 noon and in summer at 9.45 A.M. Probationers have a fortnight's holiday every six months; day charge nurses three weeks every six months; and the night charge nurse a fortnight at the end of every three months."

"The holidays are longer than usual," continued the Superintendent, "because the work is arduous, many of the patients being unable to do anything for themselves. That of the night charge nurse is exceptionally so. A great many of the deaths occur in the night. There is an average of quite a hundred deaths in the year."

"But has not the night charge nurse other help than that of the probationers on duty?"

"The porter, who has been here eight or nine years, can be called. There is an assistant porter, who is available when necessary. They move the patients up and down stairs. Of course, there are wardmaids—we have four—to do the hard work."

### The Nurses' Home.

As we passed through the commodious and nicely appointed mess-room, and along the covered passage to the

The Nurses at Friedenheim.

Nurses' Home, I remarked that the wards, and even many of the patients, seemed bright and cheerful.

"We try," rejoined the Superintendent, "to maintain a bright atmosphere always, and in summer most of the patients walk or lie about in the garden. With regard to the Nurses' Home, which we are entering, and which was opened in June 1902, we struggled on here for ten years without one, until at last I told the Council that we could not stand it any longer. It was an exceeding disadvantage to the nurses, who never got away from their surroundings. The matron's sitting-room in the hospital was their sitting-room, the room I now keep for the use of sick nurses was divided into cubicles, and the whole organisation of the place suffered. Now, they have not only the home, but a private garden attached to it right away from the hospital and its garden."

"Has each nurse a separate bedroom?" I inquired, as we inspected a comfortably furnished room with every convenience and a fireplace.

"Yes, and this is similar to the others. Every one has a fireplace. The night nurses are quite shut off on the second floor. As you see, the large general sitting-room contains a handsome piano and a harmonium—both of which were presents—as well as plenty of lounge and easy chairs. With respect to outside recreations, people are kind in sending tickets for concerts."

An Indispensable Qualification.

"There is one other question. I conclude that personal character has to be most carefully considered?"

"It is of vital importance. We need, in a special degree, nurses who possess, not so much social education, as genuine refinement. I can assure you that it is wonderful to observe how even the roughest patient usually gets softened and refined after a stay here of two or three weeks. But just as one nurse with refinement can raise the tone, so one without it can lower the tone. We do think, in short, that in a hospital like this it is not enough that a nurse should not be merely indifferent to religion; she must have a real love for it herself."

### Monthly Nursing in Germany, Holland, and Hungary.

It seems the custom in some of the Continental countries for the lady to engage a doctor to attend her as soon as she is pregnant. In the large towns she engages an obstetrician who does no general practice; but in the country an ordinary practitioner secured, unless the lady is very rich, and then a "specialist" comes from town. Having got her a doctor, he pays occasional visits up to the confinement, after which he calls daily until the lady is up and about. In Holland if there is anything of the nature of an operation or a need for medical treatment, another medical man calls regularly, and, if the family is well off and the child is ill, a third children's doctor is called in, who will pay a visit each day. The "specialist" has to do with nothing but the confinement itself. During the nine months the specialist gives the lady advice as to her own health, and instruction with regard to the expected infant.

In Germany a well-to-do lady engages, as well as the doctor, a Hebamme or midwife, who remains ten days, a wärterin or monthly nurse, also a kinderfrau or children's nurse. All these women will have passed examinations, even the kinderfrau, who must understand the different forms of food and the entire care of the infant.

The wärterin takes care of the lady entirely, but only looks after the baby during the day. The kinderfrau has charge of the baby at night, and washes and dresses it, under the supervision of the wärterin.

For the first twenty-four hours after birth the infant is not brought to the mother to feed, but it has a little sugar and water or phenal tea given it. For the next two days it is put to the breast every four hours. From 10 or 11 P.M. the baby is supposed to sleep till 5 or 6 A.M., and is not given a feed during those hours. If the baby has flatulence or pain, medicine is seldom given; castor oil never. No brandy is allowed except in cases of severe illness and by doctor's orders. The mother may have plenty of fresh fruit; sometimes she is given ℥x. phenal tea, which is very good for mother and child. If the baby has constipation a water injection, hot fomentations, rubbing the abdomen with warm oil, may be tried, or, for wind, a catheter may be inserted in the rectum to let it escape. A kinderfrau may not inject a piece of soap without the doctor's permission.

In Hungary it seems customary to keep the mother awake for three hours after the baby is born, lest she should sleep too soundly, and in all three countries the mother is encouraged to over-eat, and take large quantities of milk, which is always sterilized. She takes veal where an Englishwoman would take mutton. Mutton abroad is not nearly so good as in England, therefore instead of being considered a good article of food, it is looked upon as indigestible during confinement. Moreover, the ladies dislike it. In Hungary caraway soup is made, as it is supposed to increase the quantity and quality of the milk. Some German doctors do not consider it wise for either the mother or the baby for the former to nurse it lying down in bed; so as soon as the mother is able to sit up she is propped up in bed with pillows, and feeds it in that position. This may be because the baby usually lies on a pillow.

If the mothers are unable or unwilling to nurse their infants themselves, then a good healthy young woman must be chosen as wet nurse. These women are more easily obtained than in England, where a wet nurse is the last resource to fall back on. Failing that, the child must be brought up on bottles. The usual bottles are those of Professor Soxhlett, by whose apparatus the milk and water is sterilized. The strength of the bottle is almost the same as ours—namely, $\frac{1}{3}$ milk, $\frac{2}{3}$ water, ʒj. sugar of milk. No cream is ever given, as it is considered too heavy. Professor Soxhlett's apparatus is most convenient, and the babies thrive on the food treated in this way. As a general rule seven bottles are the utmost number given in the 24 hours. If the baby suffers from flatulency, some phenal tea is put into one of the bottles.

In a German town all the milk is brought to one or two large depôts, from which the milk shops get their milk, for there are not a number of small dairies each having their own shops, as in England. All milk for drinking-purposes is sterilized, and a foreign lady would be horrified at using anything else.

The clothing of the baby is very different from that of an English child. After the navel cord has separated, the German nurse straps the little abdomen with some plaster, in case the navel should protrude or the child rupture. It wears a knitted cotton jacket, flannel binder, and sometimes a cotton one over it. The napkins are put on in the usual way, then a small flannel square, then a large linen diaper is put on straight, so that one edge folds over the top of the pretty outside flannel, and all pinned round the armpits, so that the little arms are free. The end is pinned over the feet, and the child fastened to its pillow. The German babies wear caps and bibs, but they do not have their legs bound, as in Hungary.

The washing of the baby is also different. The "Wickeltisch" is like a chest of drawers with a railing round the top, about 10 in. high. The front lets down while the child is being washed. On the top is a firm mattress with washable cover. In Hungary a pillow stuffed with straw is used, so that a hole can be made in it. The nurse stands to wash the child. On this wickeltisch the child is undressed, then washed in the bath, taken out on to the wickeltisch, and well rubbed and dried with hot towels and quickly dressed. The child is turned about more than by the English method. It looks so droll to see the little thing trying to crawl on "all fours" and lift its tiny head.

There is not much difference in the cots, only blankets are not used, but either a small feather bed or a thick-padded quilt, covered with silk, on to which a linen covering with lace border is buttoned. The pillow is covered with silk to match, over which the lace slip is drawn. It looks very dainty.

In Holland at the end of three weeks, when the mother is quite well, she holds a reception of her lady friends. The pretty cot is placed in the drawing-room with its occupant in it on show. The cot is trimmed with pale-blue ribbons if the baby is a girl, and with pink if a boy. When the tea is handed round it is accompanied by tiny sweets of a special sort—white for a girl and pink for a boy.

### Practical Hints.

**We welcome notes on practical points from nurses.**

### BED-PANS.

A few suggestions as to different ways of giving the bed-pan to very helpless patients, besides the one mentioned by the writer of the articles on "The Care and Nursing of the Insane," may be useful. In the country, when houses are often detached and standing in their own gardens there is often a great difficulty in getting any one in to help without a waste of time; therefore, one learns to manage for oneself. I have been a district nurse for thirteen years and just now I have two very old helpless women in my district, both over 70 years of age, and enormously stout, with no one to attend to them except their old husbands. So I just roll the patient on to her side, place the bed-pan in position, and bring her back on to it. On removing the pan I again push her over on to her side, so am able to manage alone, and find it much easier than trying to raise her. One of the old women has been unable to bend her knees for seven years, the other, poor soul, is too far advanced in senility to understand how. When a bed-pan is not avilable I have recourse to an old enamel flat pie-dish, or the soap-dish belonging to an old-fashioned toilet-set. I find a cleaner and easier way of emptying them is to put a layer of paper at the bottom before use. In a country place where water is scarce, and in the houses of the very poor a nurse has to use a little common sense and not always just what she did in hospital. Instead of a flannel covering for the pan which, personally, I dislike, I pour a little hot water over the pan, which warms it nicely if no fire is in the room.

### To Nurses.

**We invite contributions from any of our readers, and shall be glad to pay for "Notes on News from the Nursing World," "Incidents in a Nurse's Life," or for articles describing nursing experiences at home or abroad dealing with any nursing question from an original point of view, according to length. The minimum payment is 5s. Contributions on topical subjects are specially welcome. Notices of appointments, letters, entertainments, presentations, and deaths are not paid for, but we are always glad to receive them. All rejected manuscripts are returned in due course, and all payments for manuscripts used are made as early as possible after the beginning of each quarter.**

### A Retrogression in Edinburgh.

With reference to the letter under this heading, which bore the signature "One Interested in the Improvement of Mental Nursing," and appeared in The Hospital of December 1, we have received the following letter from Messrs. Thomson, Dickson & Shaw, of 1 Thistle Court, Edinburgh, solicitors for Sir John Batty Tuke, M.B. :—

1 Thistle Court, Edinburgh, December 17.

The Secretary,
The Scientific Press, Ltd.,
28 Southampton Street, Strand, London.

Sir,—Sir John Batty Tuke's attention has been called to a paragraph in The Hospital of 1st inst. headed "A Retrogression in Edinburgh," which is false and libellous, and we are surprised that it should have appeared in your columns.

The institution there referred to as Mavisbank has recently been amalgamated with Saughton Hall Asylum. Sir John Batty Tuke is medical director of the amalgamated institution (known as "New Saughtonhall"), and Dr. J. Batty Tuke is medical superintendent.

We enclose a copy of the only document of the nature of a prospectus which has been issued by or on behalf of the new institution, and in which the words quoted in your correspondent's article do not appear, and further we have to state that all the nurses have served in similar institutions or in general hospitals for periods ranging from one and a half to seven years, and that they are drawn from a highly respectable class of society. The insinuation regarding dividends is calumnious.

In these circumstances we request that you will give prominence in your next two issues to an ample apology, the terms of which we will adjust with you; and that you also give us the name and address of your correspondent.

Your obedient servants,
Thomson, Dickson and Shaw.

We publish this letter at once without delaying it to arrange with Messrs. Thomson, Dickson and Shaw the form of the apology for which they ask. The document sent to us by them does not contain the expressions which appeared in our correspondent's letter as quotations, nor does it contain anything in the nature of a guarantee of dividends.

We have therefore written to the writer of the letter complained of, calling for proofs of the statements in question.

Without waiting for the reply, we express our sincere regret that anything defamatory or inaccurate concerning Sir John Batty Tuke should have appeared in our columns, and we make to him an unreserved apology for the publication of a letter reflecting unfavourably on one of his institutions, which we received and published in good faith and without any idea that the writer's statements could be challenged.

### Everybody's Opinion.

### POLITENESS.

"R. A." writes: How is it that nurses in general are wanting in courtesy to one another. I cannot understand it—they do not like to be classed with "porters." Then why is it that they forget to treat one another kindly and politely? I have worked very hard in my time. My training, I am quite certain, was in the hardest school with the severest rules in London, but I was made sister and then matron. I have worked both as district and as private nurse, and have remembered, I think, what my father told me as a child, "Politeness was due to a tramp." I am a gentlewoman by birth, and try my best to treat nurses as I wish to be treated. I am not surprised that private patients complain, and their maids hate the sight of nurses. If they were all treated kindly and with consideration I feel sure that we should be looked upon as a comfort rather than as plagues in the household. I will give a simple instance. Since you opened your excellent "Sale and Exchange" column I have answered constantly and received no reply. If one can advertise, cannot she reply either by letter or by postcard "Sold"? To wait and wait means anxiety to the person who replies, especially to the one isolated—far from station and shops. Cannot we all try to be a little more civil to one another? Then, perhaps, we should not get classed with "porters," who, by the by, often have kindly ways and certainly remember "Manners maketh man."

### A PROBATIONER'S POINT OF VIEW.

"P. O. B." writes: I have been a nurse now for some time, though, perhaps, that is rather a misleading statement—it sounds as if I had been nursing for several years. But I am speaking from a probationer's point of view. For "pros." do have points of view, and even opinions on different matters, though, of course, hospital etiquette forbids them such luxuries. Perhaps that is why they are so plentiful and so decided. I have been at this hospital long enough to know whether I am going to like it or not. As far as that goes I liked it from the very beginning. There is a strange sort of fascination about the life, apart from the work itself. But the drawbacks and disadvantages which the training entails are very great and might have led some of us to look upon ourselves as heroines and that sort of thing, if matron had not told us we were nothing of the kind; and then the compensations are on a large scale. The things a nurse has to do, and thinks almost nothing of, after the first few dreadful days, would astonish her friends in the outside world. The sweeping and dusting, cleaning and polishing are the chief duties of the "pro."—but while she is doing this she can keep her eyes and ears open and learn a lot. We are all like Dickens' character, the dancing master, I forget his name, "we do our best to polish, polish, polish." But our best generally seems to be dreadfully low down in the scale, according to those whom Fate and Father Time have placed above us. But in spite of the scrubbing and cleaning, the amount and character of which would have horrified me less than a year ago, I have never been happier in my life, and I should be heart-broken if I had to give it up. As a beginner, it seems to me that your whole outlook depends upon your staff nurse. If she is nice, life is worth living. But if she is not, your existence is simply a misery. The very brooms and brushes assume a different aspect, the dusters always look dirty, and the brasses will not shine. Life is a burden not worth bearing. I have worked under such a one, fortunately not at the beginning. But my experience is that there are very few like that. If you do your best, unless you are unusually slow and stupid, there will not be very much to find fault with, and if they see you putting your whole heart into your work when you do make mistakes they will not blame you much more than you deserve.

### A NURSE'S LACK OF DECISION.

"A Disappointed Sister" writes: Through your columns I would like to enlighten some fellow-nurses upon the inconvenience it is possible for some nurses to suffer by another's lack of decision. A selected candidate was appointed by our Board of Guardians to the post of Nurse. There was nothing to lead the Guardians to believe that the candidate was not satisfied. Imagine our great disappointment when, a day or two afterwards, the nurse wrote to the clerk declining the post. Nothing further could be done until the following Board day, we in the meantime being very handicapped for help. The Guardians decided to advertise again. Although it is more than a month since the late nurse vacated her post, I am still at work single-handed in a block of forty-four or forty-five patients. There is one day nurse and one night nurse, and as the female block is some distance from the male it is not possible for the nurses to relieve each other, except at the risk of leaving unattended patients who are very ill, fatuous, or epileptic. We can now only go off duty when the night nurses can relieve us. This confinement has extended over a period of six weeks, and there is no prospect of relief this side of Christmas, the extra work being more or less expected of us whenever we are short-handed. I am told that a nurse declined the post some years ago, and the conditions then were nearly similar to what they are now. I cannot help thinking it is a pity that a nurse cannot keep to her decision, especially after being shown over the field of work.

### Appointments.

Jessop Hospital for Women, Sheffield.—Miss Louisa C. Harding has been appointed staff nurse. She was trained at Paddington Infirmary, where she has since been staff nurse. She has also been Queen's Nurse at Bridgwater. She holds the C.M.B. certificate.

Royal National Hospital for Consumption for Ireland, Newcastle, co. Wicklow.—Miss H. Pagan has been appointed night sister. She was trained at the Adelaide Hospital, Dublin, and has since held the post of sister of the enteric and diphtheria blocks, Enfield Hospital, Middlesex, and sister at the Royal Hants and Southampton Hospital, Southampton. She also had a course of training at the Rotunda Hospital, Dublin.

Runcorn Urban District Infectious Disease Hospital. Miss Mary C. G. Chrystie has been appointed nurse matron. She was trained at the Royal Infirmary, Perth. She has since been ward sister at the Isolation Hospital, Crewe. She has also done private work, and holds the C.M.B. certificate.

Workhouse Infirmary, Milton, Portsmouth.—Miss B. M. Bryant and Miss Emma J. Carter have been appointed charge nurses. Miss Bryant was trained at Whitechapel Union Infirmary, and has since been nurse at Leicester Infirmary. Miss Carter has been trained at St. Pancras Infirmary.

### Presentations.

Christchurch Workhouse Infirmary.—Miss L. E. Lynn, upon leaving her post as charge nurse at Christchurch Workhouse Infirmary to take up the post of superintendent nurse at the Isle of Wight Workhouse Infirmary, was presented with a travelling-bag by the medical officer and nursing staff and with a travelling-clock by the Master and Matron.

### Our Christmas Distribution.

The following letters have been received by us from the recipients of parcels of garments which we were, through the kindness of our readers, able to distribute:—

The Matron of the Metropolitan Hospital, Kingsland Road, N.E., writes: I am writing to thank you and your readers very heartily for your most kind gift of toys, clothes, etc., for our patients. They are so warm and well made that many a heart will be gladdened by your kind thoughts, and they will help to brighten Christmas Day for the very poor who have so little to cheer and help them.

The Matron of the City of London Hospital for Diseases of the Chest, Victoria Park, E., writes: It is with many grateful thanks I have to write to you for so kindly remembering us again amongst your Christmas presents this year, as I assure you such clothing is indeed very acceptable for our poor patients this cold weather.

The Matron of the National Hospital for Diseases of the Heart, Soho Square, W., writes: I am most grateful for the gift of useful clothing sent for the patients to-day. They will be a great help during the coming season for the several poor children in the hospital at the present time.

The Secretary of St. Mary's Hospital for Women and Children, Plaistow, E., writes: Please accept my warmest thanks for your kindly present of garments and toys, which is much appreciated.

### Notes and Queries.

**REGULATIONS.**

**The Editor is always willing to answer in this column, without any fee, all reasonable questions, as soon as possible.**

**But the following rules must be carefully observed.**

1. **Every communication must be accompanied by the name and address of the writer.**
2. **The question must always bear upon nursing, directly or indirectly.**

**If an answer is required by letter a fee of half-a-crown must be enclosed with the note containing the inquiry.**

*Nursing Home Abroad.*

(148) Can you give me the address of the Anglo-American nursing home in Rome?—*Shrewsbury.*

It is 265 Via Nomentana, Rome.

*Co-operative Societies.*

(149) Can you tell me which are the best co-operative societies in London to belong to?—*Reader.*

The Nurses' Co-operative, 8 New Cavendish Street, W., is the principal co-operative society in London. There are others in "Burdett's Hospital and Charities," under the head of "Nursing Institutions," published by the Scientific Press, 28 and 29 Southampton Street, Strand, London, W.C.

*Rural Midwives.*

(150) I am a fully-trained nurse; will you kindly tell me if it is possible, in return for my services, to obtain a midwifery training?—*Hexham.*

If you write to the Rural Midwives' Association, 47 Victoria Street, S.W., or the Association for Promoting the Training of Midwives, Dacre House, Dean Farrar Street, Westminster, S.W., they will be able to give you the information you want.

*Epilepsy.*

(151) Can you kindly suggest a home for a young woman, an epileptic, in a weak state of health?—*Jones.*

Write to the Secretary of the Meath Home of Comfort for Female Epileptics, Westbrook, Godalming.

*Masseuse.*

(152) Will you kindly tell me where in London I could receive instruction in massage; and is the work of a masseuse lighter than that of a nurse?—*Rizza.*

The Incorporated Society of Trained Masseuses, Office 12 Buckingham Street, Strand, W.C., would probably advise you. The work of a masseuse requires a great deal of physical strength, but the strain is not so continuous as that imposed upon a nurse.

*Lunacy.*

(153) Can you tell me of an institution or asylum where a trained nurse can be received as a patient at the rate of £1; certified case?—*Perplexed.*

You could probably be received as a paying patient at St. Luke's Hospital for Lunatics, Old Street, E.C., or some of the London County asylums receive private patients for the cost you state. You will find a list in "Burdett's Hospitals and Charities," price 6s. 4d., post free, The Scientific Press, 28 and 29 Southampton Street, Strand, London, W.C.

*Maternity Nurse.*

(154) Can you tell me of any training school in or near Glasgow which would give midwifery training free? I have fever and private experience.—*Glasgow.*

Write to the Matron, Glasgow Sick Poor and Private Nursing Association. She may be able to advise or help you.

*Infant Life.*

(155) Can you give me the address of the Society for Infant Life Preservation?—*Hythe.*

We do not know of a society of that name. Perhaps you mean the society formed in connection with Marylebone. If so, write for particulars to Eric Pritchard, Esq., M.D., The St. Marylebone Dispensary, N.W.

**Handbooks for Nurses.**

| | Post Free. |
|---|---|
| "A Handbook for Nurses." (Dr. J. K. Watson) ... | 5s. 4d. |
| "Nurses' Pronouncing Dictionary of Medical Terms" | 2s. 0d. |
| "Art of Massage." (Creighton Hale) ... ... ... | 6s. 0d. |
| "Surgical Bandaging and Dressings." (Johnson Smith.) ... ... ... | 2s. 0d. |
| "Hints on Tropical Fevers." (Sister Pollard.) ... | 1s. 8d. |

Of all booksellers or of The Scientific Press, Limited, 28 & 29 Southampton Street, Strand, London, W.C.

### For Reading to the Sick.

"NOT AS I WILL."

"Not as I will:"—the sound grows sweet
Each time my lips the words repeat.
"Not as I will:"—the darkness feels
More safe than light, when this thought steals
Like whispered voice to calm and bless
All unrest and all loneliness.
"Not as I will," because the One
Who loved us first and best has gone
Before us on the road, and still
For us must all his love fulfil,—
"Not as we will."

*Anon.*

Christ with us in every dark path, in every lonely way. We are clearly taught that the love of God never fails His children, that it is as true and tender in times of affliction as it is in times of gladness, that it is the same when blessings are taken away as when they are given. We know that all things work together for good to them that love God. It is made plain in the Scriptures that no tribulation can harm us if we abide in Christ, that we shall be preserved blameless through the most terrible trials, if our faith in Christ does not fail. Many of life's events are full of mystery—we cannot understand them, nor can we see how they are consistent with God's love and wisdom. But we have the most positive assurance that some time we shall understand, and that in everything we shall see Divine goodness.

In our Lord's experience in Gethsemane we have another example of a like working of prayer. The cup for whose taking away the Holy Sufferer pleaded with strong crying and tears was not withdrawn, and yet the anguish of his heart grew less and less intense until we hear the word of victory, "The cup which the Father hath given Me, shall I not drink it?" The supplication availed in its working, not in saving Him from the bitter experiences on which He was entering, but in the giving of help which enabled Him to pass through all the terrible fifteen hours that followed, without murmuring.

Not only should we endure affliction victoriously, sustained by Christ, but we should emerge from it ready for better service and for greater usefulness than ever before. We are told that Jesus was made perfect through suffering. He learned in His own experience of sorrow how to sympathise with His people in their sorrows and how to comfort them. One of the reasons for trouble is that in it we may be prepared for helping others in their troubles. Sorrow is a school, and we meet it as we should only when we learn the lessons and go out fitted for being a richer blessing in the world.—*Dr. J. R. Miller.*

If anyone would tell you the shortest, surest way to all happiness and all perfection, he must tell you to make it a rule to yourself to thank and praise God for everything that happens to you. For it is certain that whatever seeming calamity happens to you, if you thank and praise God for it, you turn it into a blessing. Could you, therefore, work miracles, you could not do more for yourself than by this thankful spirit; for it heals with a word spoken, and turns all that it touches into happiness.—*Wm. Law.*

## Figures and Tables

**Figure f1:**
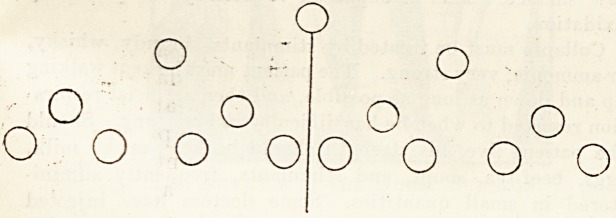


**Figure f2:**
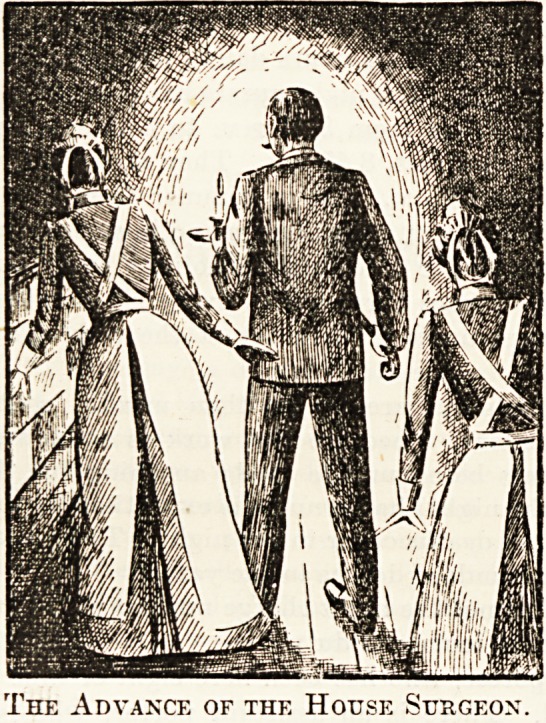


**Figure f3:**
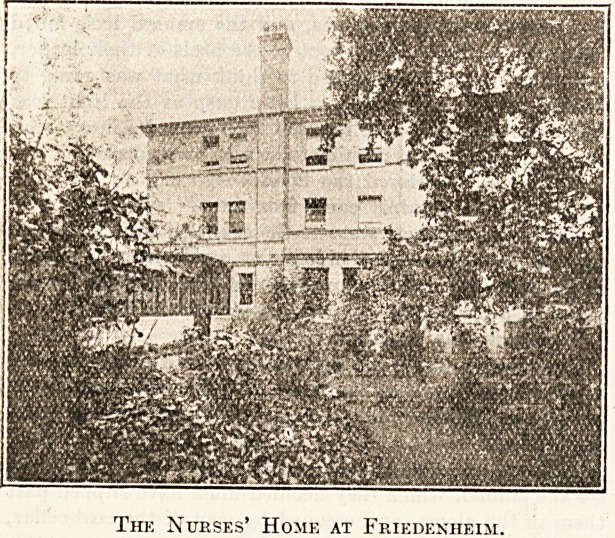


**Figure f4:**